# CareBuddy: A Multi-Agent Conversational AI for Alzheimer’s Care and Assistance

**DOI:** 10.1093/geroni/igaf122.3554

**Published:** 2025-12-31

**Authors:** Wordh Ul Hasan, Ayanle Aideed, Kimia Tuz Zaman, Juan Li

**Affiliations:** University of Minnesota Crookston, Fargo, North Dakota, United States; North Dakota State University, North Dakota State University, North Dakota, United States; North Dakota State University, Fargo, North Dakota, United States; North Dakota State University, Fargo, North Dakota, United States

## Abstract

Managing Alzheimer’s disease presents daily challenges for both the individuals living with the condition and their informal caregivers. While conversational AI offers potential support, traditional single-agent systems often lack the specialization and context-awareness required for comprehensive care. This study introduces and evaluates CareBuddy, a modular, multi-agent conversational AI system designed to provide proactive, personalized assistance to both persons with Alzheimer’s and their caregivers. CareBuddy features a layered architecture with specialized agents for medical inquiries, appointment scheduling, meal planning, and reminders, coordinated by a central orchestrator. A mixed-methods usability study was conducted with 20 participants—including family caregivers, older adults with and without early-stage memory impairment, and healthcare professionals—to assess effectiveness and usability. Results demonstrated high task completion rates and user satisfaction, with 85% of users rating appointment scheduling 5/5 and 90% rating grocery planning 4 or 5. The system significantly reduced task completion time and cognitive load. The findings indicate that a modular, context-aware multi-agent AI framework can substantially improve daily management and confidence for the entire care dyad, holding promise for integration into broader healthcare platforms.

